# Supplementary material for: The proximal lipid phase of PI3K signaling is confined to the plasma membrane
Source: bioRxiv. 2026 May 12:2026.05.08.723799. Preprint. [Version 1] doi: 10.64898/2026.05.08.723799 (PMC13193016; doi:10.64898/2026.05.08.723799)
Supplement: Supplement 1 [file NIHPP2026.05.08.723799v1-supplement-1.pdf]

bioRxiv preprint doi: https://doi.org/10.64898/2026.05.08.723799; this version posted May 12, 2026. The copyright holder for this preprint (which was not certified by peer review) is the author/funder, who has granted bioRxiv a license to display the preprint in perpetuity. It is made available under a CC-BY 4.0 International license.

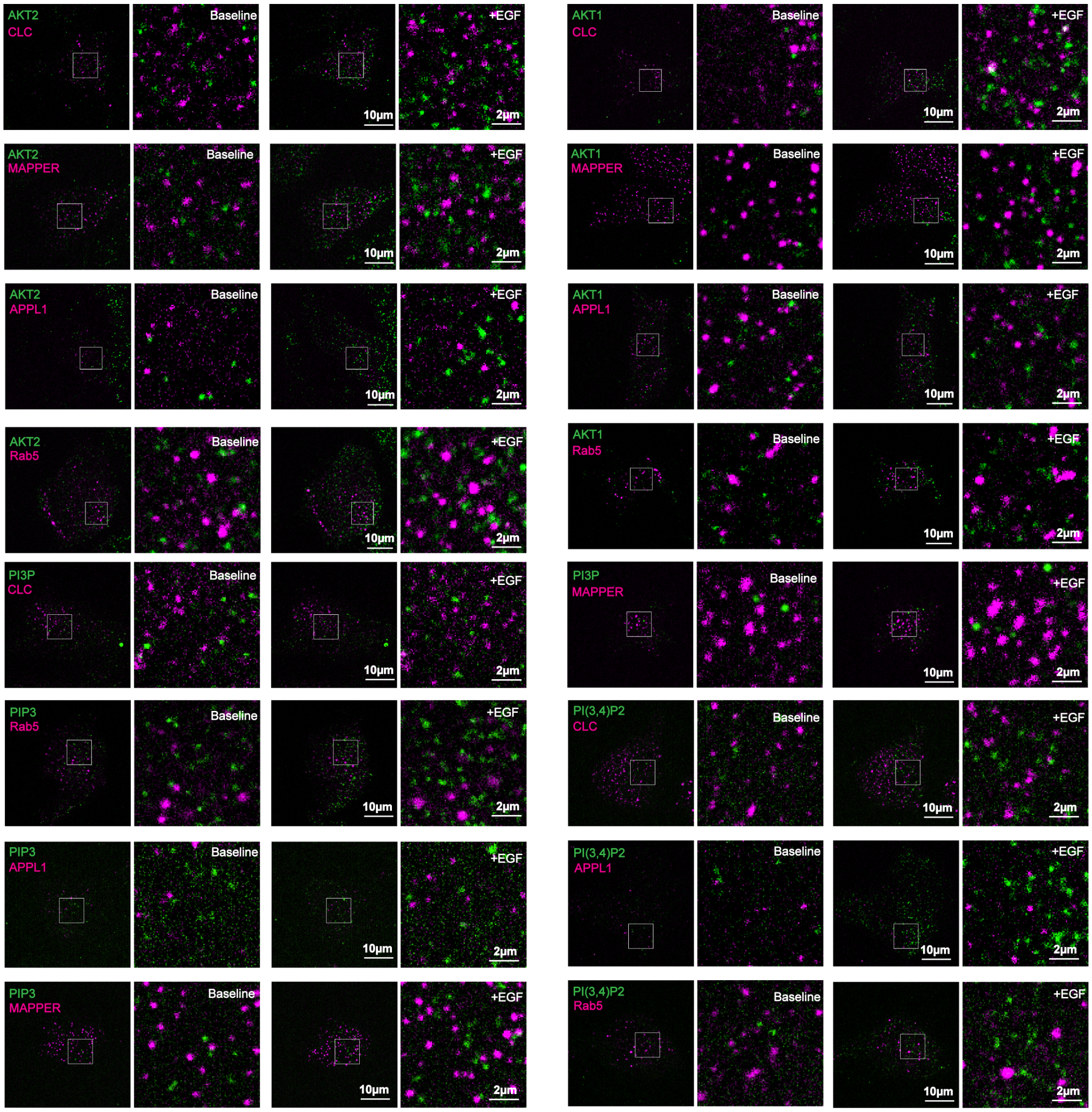

FIGURE S1: Representative images of endogenous NG2-tagged AKT1 or AKT2 or the indicated lipid biosensors, together with organelle markers pre- and post-EGF administration. For each image pair, the left image shows then entire cell footprint, whereas the right image shows the inset.

bioRxiv preprint doi: https://doi.org/10.64898/2026.05.08.723799; this version posted May 12, 2026. The copyright holder for this preprint (which was not certified by peer review) is the author/funder, who has granted bioRxiv a license to display the preprint in perpetuity. It is made available under a CC-BY 4.0 International license.

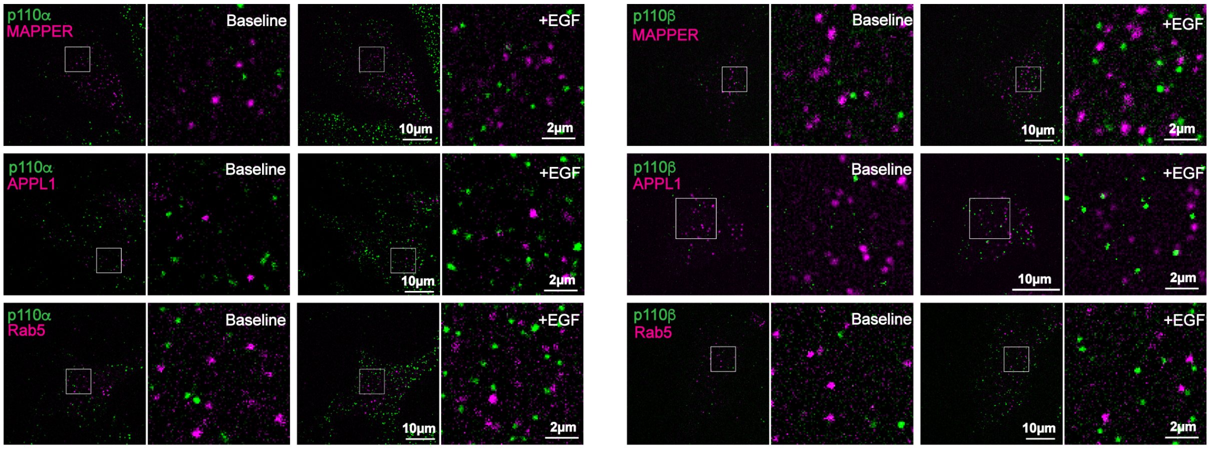

FIGURE S2: Representative images of endogenous mNeonGreen-tagged p110α or p110β, together with organelle markers pre- and post-EGF administration. For each image pair, the left image shows then entire cell footprint, whereas the right image shows the inset.

bioRxiv preprint doi: https://doi.org/10.64898/2026.05.08.723799; this version posted May 12, 2026. The copyright holder for this preprint (which was not certified by peer review) is the author/funder, who has granted bioRxiv a license to display the preprint in perpetuity. It is made available under a CC-BY 4.0 International license.

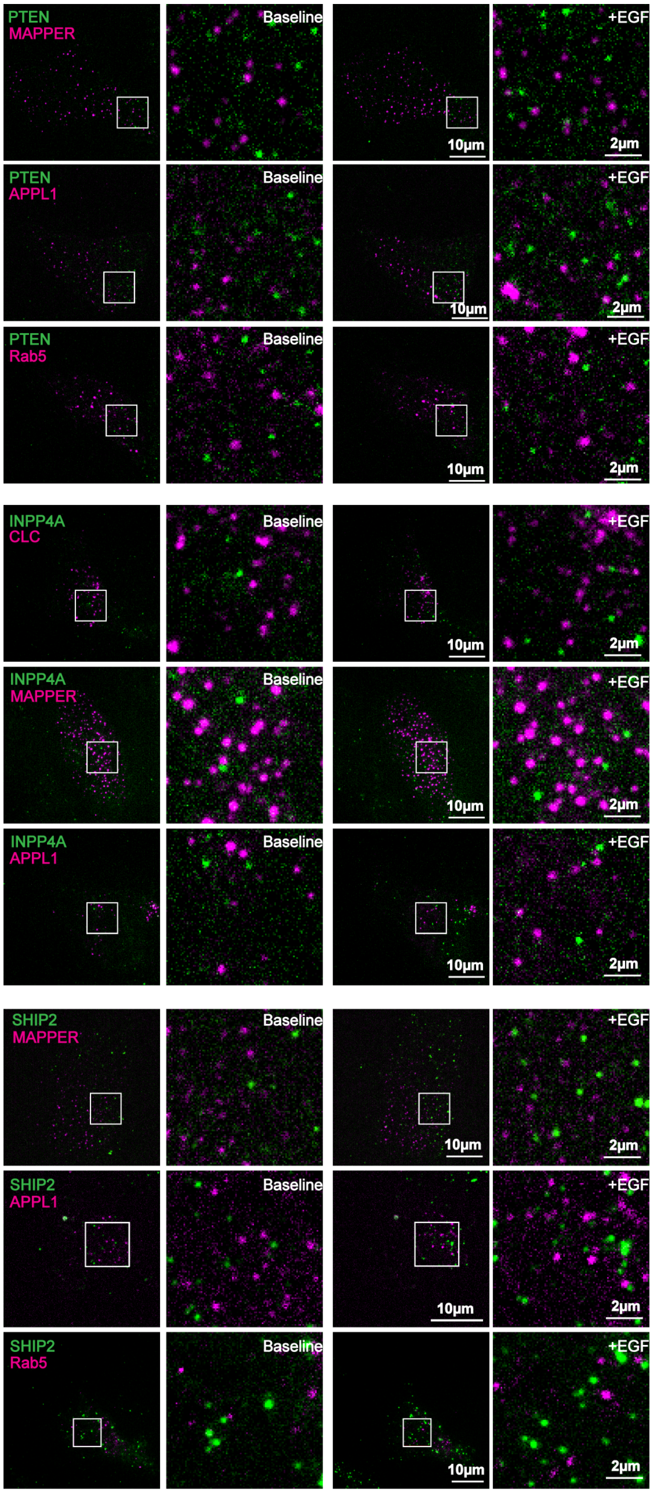

FIGURE S3: Representative images of endogenous tagged PTEN, INPP4A or SHIP2, together with organelle markers pre- and post-EGF administration. For each image pair, the left image shows then entire cell footprint, whereas the right image shows the inset.
